# Development and Usability Testing of an mHealth Tool for Trauma-Informed Prevention of Substance Use, HIV Acquisition, and Risky Sexual Behaviors Among Adolescents: Mixed Methods Study

**DOI:** 10.2196/52835

**Published:** 2024-01-18

**Authors:** Carla Kmett Danielson, Angela Moreland, Austin Hahn, Devin Banks, Kenneth J Ruggiero

**Affiliations:** 1 Department of Psychiatry & Behavioral Science Medical University of South Carolina Charleston, SC United States; 2 Department of Psychology University of Missouri-Saint Louis Saint Louis, MO United States; 3 College of Nursing Medical University of South Carolina Charleston, SC United States

**Keywords:** traumatic stress, prevention, substance use, HIV, qualitative methods, adolescents, mobile phone

## Abstract

**Background:**

Youth who experience traumatic events are at a substantially higher risk of engaging in substance use and sexual risk behaviors and problems (eg, HIV acquisition) than their non–trauma-exposed counterparts. Evidence-based substance use and risky sexual behavior prevention may reduce the risk of these outcomes. Trauma-focused mental health treatment provides a window of opportunity for the implementation of such preventive work with these youth. However, overburdened clinicians face challenges in adding prevention content while implementing evidence-based treatments. Mobile health (mHealth) tools can help reduce this burden in delivering prevention curricula. Trauma-Informed Prevention for Substance Use and Risky Sexual Behavior (TIPS) is an mHealth app that was developed to aid trauma-focused cognitive behavioral therapy (TF-CBT) clinicians in the implementation of an evidence-based risk behavior prevention curriculum.

**Objective:**

The goal of this paper is to describe the rationale for and development of the TIPS app and present the results of a mixed methods approach for the initial evaluation of its usability.

**Methods:**

Participants included clinicians (n=11), adolescents (n=11), and caregivers (n=10) who completed qualitative interviews and an adapted version of the Website Analysis and Measurement Inventory.

**Results:**

In total, 4 overarching themes emerged from the participants’ answers to the qualitative interview questions, demonstrating a generally positive response to the app. The themes were (1) strength of app content, (2) suggestions about app content, (3) esthetics and usability, and (4) benefits to the patient and session implementation. Clinicians, adolescents, and caregivers all agreed that the content was very relevant to adolescents and used examples and language that adolescents could relate to. All 3 groups also discussed that the content was comprehensive and addressed issues often faced by adolescents. All 3 groups of users made suggestions about the esthetics, which mostly comprised suggestions to change the font, color, or pictures within the app. Of all the groups, adolescents were most positive about the esthetics and usability of the app. Results from the Website Analysis and Measurement Inventory further illustrated the users’ favorable reaction to the TIPS app, with 100% (11/11) of clinicians, 100% (10/10) of caregivers, and most adolescents (7/11, 64%) selecting *strongly agree* or *somewhat agree* to the following statement: “This app has much that is of interest to me.” Adolescents generally found the app easier to use than did caregivers and clinicians.

**Conclusions:**

The TIPS app shows promise as an mHealth tool for TF-CBT clinicians to integrate evidence-based substance use, risky sexual behavior, and HIV prevention during treatment. Future research, including a randomized controlled trial comparing TF-CBT implementation with and without the inclusion of the app, is necessary to evaluate the feasibility and efficacy of the app in reducing the risk of substance use and risky sexual behavior among trauma-exposed adolescents.

**Trial Registration:**

ClinicalTrials.gov NCT03710720; https://clinicaltrials.gov/study/NCT03710720

## Introduction

### Background

Children and adolescents who experience traumatic events are at a substantially higher risk of engaging in sexual risk behaviors and substance use than their non–trauma-exposed counterparts [1,2]. Early traumatic experiences also have long-term effects on behavioral health as youth exposed to trauma are more likely than their nonexposed peers to develop substance use disorders [3,4] and experience unexpected pregnancy and sexually transmitted infections (STIs) [5,6], including increased risk of HIV acquisition [7,8]. Models addressing the link between trauma exposure and risk behavior have suggested that, although these behaviors are multidetermined, trauma exposure plays an important role by manifesting impairments in affect regulation, impulse control, identity development, and socialization [2,9].

Given the strong relationship between trauma exposure and problematic behavioral health outcomes, preventing morbidity related to substance use and sexual risk behavior may be best addressed from a trauma-informed lens. That is, an important prevention approach may involve addressing risk behavior in the context of trauma-focused assessment and treatment. However, data suggest that clinicians are often reluctant to integrate interventions related to substance use in the context of trauma treatment [10] because of clinical, systemic, and training barriers and limited resources (including limited support) relative to caseload demands [10-13]. Given the need for substance use and risky sexual behavior prevention for trauma-exposed adolescents—and to combat these barriers and demands for time and resources—clinicians may benefit from a structured tool to help efficiently implement evidence-based prevention strategies for risk behaviors common among trauma-exposed youth [14]. Such a tool may be best delivered via a mobile health (mHealth) approach, which can reduce the need for extensive clinician training in multiple new prevention intervention curricula, augment the formal training they do receive, and enhance or extend the effectiveness of the traditional clinical encounter [15,16]. As such, clinicians would be significantly more likely to deliver substance use prevention if a ready-made mHealth tool were available that helped them deliver it efficiently and accurately. This study describes the development and feasibility evaluation of an mHealth app designed to supplement trauma treatment among adolescents, targeting prevention of substance use, sexual risk behavior, and associated health consequences (eg, HIV).

### Adolescent Trauma Treatment as an Opportunity for Prevention

The gold-standard treatment for addressing adolescent trauma is trauma-focused cognitive behavioral therapy (TF-CBT) [17,18]. With >20 completed randomized controlled trials (RCTs)—including international studies—supporting its effectiveness in addressing a range of mental health problems and improving functioning among trauma-exposed youth, TF-CBT has received the highest ranking for empirical support from professional organizations and federal agencies [19-38]. TF-CBT has achieved widespread dissemination through numerous implementation efforts, including comprehensive training programs (eg, community-based learned collaboratives), educational materials, and collaborations with mental health organizations, ensuring its accessibility and use by therapists and clinicians across various regions and populations. In fact, the widespread utility of TF-CBT is exemplified by the staggering number of clinicians who have accessed TF-CBT Web 1.0 and 2.0 (original and updated versions of the TF-CBT web-based learning course); as of August 31, 2023, a total of 496,061 clinicians worldwide are registered users of the web-based training in the TF-CBT model, and 305,120 clinicians have completed the training [39]. Modules of TF-CBT, including psychoeducation and enhancing safety, may include general psychoeducation about sex and sexual revictimization risk reduction; however, information and skill development specific to HIV and STIs, pregnancy, and healthy dating and sexual decision-making are not detailed in the model manual or training. Similarly, skill development for preventing substance use problems that extends beyond psychoeducation and helps translate this knowledge into skills is not formally or typically incorporated into TF-CBT, limiting the potential impact of these psychoeducation modules. In other words, these clinicians do not systematically receive the training and support needed to feel confident in their delivery of psychoeducation content or in the ways to translate this education into skills (eg, realistic refusal skills) [10].

Although clinicians can informally incorporate risk behavior topics into their implementation, the data suggest that this is uncommon. A national survey of mental health clinicians found that providers feel ill-equipped to address topics of substance use and sexual risk behavior when treating trauma-exposed adolescents even when trained in trauma-informed treatment models [10]. Most clinicians did not report receiving formal training for addressing substance use disorder (54%) or sexual risk behavior (67%), suggesting that this reluctance may be related to deficits in training on these topics [10]. These data reflect a global lack of training in evidence-based practice for adolescent prevention, which results in limited translation and accessibility despite the availability of numerous efficacious preventive interventions [40]. In summary, although effective sexual risk and substance use prevention interventions are available, there is a significant gap in the implementation of these interventions even among those at high risk of such behaviors because of trauma exposure.

### mHealth as a Viable Approach to Address Prevention

The availability of mobile technology has increased dramatically over the past decade, with 85% of Americans reporting smartphone ownership in 2022 compared with 35% in 2011 [41,42] and 95% of American adolescents reporting smartphone access [43,44]. As mobile technology has rapidly developed, so have health care approaches that leverage mHealth—the use and development of mobile technology, including mobile apps, to improve health care [16,45]. Within mental and behavioral health care, mHealth interventions are diverse, targeting different stages of treatment from education and engagement to the maintenance of treatment gains [16]. mHealth administrations are also diverse and may be stand-alone, client-led interventions or supplements to traditional clinician-facing treatments [16].

Most of the extant mHealth approaches to behavioral health among adolescents are stand-alone interventions rather than supplements to face-to-face treatment [46]. Several stand-alone treatments have shown strong feasibility and efficacy for both primary and secondary prevention of substance use and sexual risk behavior [47-50]. However, most stand-alone behavioral interventions for adolescents lack a theoretical framework in their design and show inconsistent efficacy [46,51]. Furthermore, although data suggest that digital mental health tools delivered in real-world contexts are more likely to be accessed than professional services, they are less likely to be delivered with a sufficient therapeutic dose [52]. These limitations are compounded by clinician- and patient-reported barriers to stand-alone treatments, including the lack of personalization [51,53], lack of privacy and security with regard to sensitive behavioral topics [53,54], limited follow-up [51], and attrition and low completion rates [54,55]. Researchers have suggested that these barriers could be overcome with more support and involvement from clinicians [53]. Accordingly, behavioral health treatments that blend face-to-face and technology-based approaches have been found to save clinician time, demonstrate lower dropout rates, and lead to better treatment outcomes among adolescents and young adults [56,57].

With regard to trauma treatment, 3 popular mHealth apps have emerged for use with adults: Posttraumatic Stress Disorder (PTSD) Coach, Cognitive Processing Therapy Coach, and Prolonged Exposure Coach [58]. The most widely used of these is PTSD Coach, a stand-alone treatment that suffers from limitations similar to those mentioned previously [58], including high attrition and inconsistent efficacy [59]. Conversely, Cognitive Processing Therapy Coach and Prolonged Exposure Coach are supplementary to existing evidence-based treatments for PTSD. Although these apps have not been evaluated for efficacy via RCTs, clinician perceptions of such apps have been favorable, particularly regarding their relative advantage over exclusively face-to-face practices and compatibility with clinicians’ needs [60]. In addition, supplementary mHealth approaches have demonstrated effectiveness in the treatment of comorbid trauma conditions (eg, panic disorder, anxiety, and depression) [61], including comorbid PTSD and substance use [62].

Despite evidence of the feasibility and effectiveness of mHealth interventions in trauma treatment among adults, mHealth approaches to trauma treatment among adolescents are limited. In total, 3 stand-alone prevention approaches for adolescents exposed to acute trauma have demonstrated effectiveness in reducing persistent trauma symptoms, depressive symptoms, and behavioral problems [63-65]. Although no studies have examined the effectiveness of supplementary mHealth approaches for adolescent trauma treatment, the perspectives of trauma-focused clinicians indicate that these approaches would be feasible and useful. For example, Orengo-Aguayo et al [66] found that 96% to 100% of surveyed TF-CBT providers reported that an mHealth supplement would be helpful for enhancing TF-CBT components, extending coping skill development, and improving out-of-session practice among adolescents and families. Clinicians, patients, and families have also responded favorably to pilot versions of a supplemental Apple iPad–based app designed to improve patient engagement and provider fidelity in-session during TF-CBT [67,68]. Thus, mHealth supplements to adolescent trauma treatment may be a feasible and acceptable way to extend and improve behavioral health prevention among adolescents.

### This Study

The purpose of this paper is to report on the development and perceived usability of Trauma-Informed Prevention for Substance Use and Risky Sexual Behavior (TIPS), a novel mHealth tablet-based app designed to supplement trauma treatment targeting the prevention of adolescent risk behavior (trial registration: ClinicalTrials.gov NCT03710720). We used a mixed methods approach based on qualitative interviews and quantitative ratings to assess the usability of the TIPS app with clinicians, adolescent patients, and their caregivers engaged in TF-CBT in a community-based outpatient clinic.

## Methods

### TIPS App Structure

**Figure 1:**
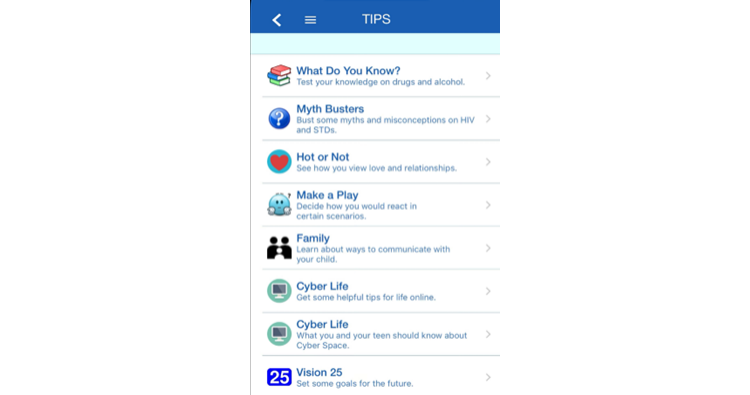
Trauma-Informed Prevention for Substance Use and Risky Sexual Behavior (TIPS) home screen (after log-in) listing the 7 topics and activities.

The first author, a national trainer in the TF-CBT model, led a small team of TF-CBT clinicians and trainees in the development of the content of the TIPS mHealth app. This included 7 total topics (Figure 1) designed to be used as psychoeducational tools for TF-CBT clinicians to implement with adolescents and caregivers throughout the TF-CBT treatment process. The tool helps clinicians assess their clients’ and caregivers’ current knowledge and comfortability surrounding topics related to risky sexual behaviors, STIs, drug use, and healthy relationships. Thus, there are 3 intended users of the app: clinicians, adolescents, and caregivers. Each user sets up a unique log-in, and the content displayed on the app is tailored to the user type. For example, *Family Check-Up* example videos demonstrating parenting skills are displayed for the caregiver but not for the youth, and a sexting decision-making activity is displayed for the youth but not for the caregivers; clinicians view both caregiver and youth content but also have unique introduction videos for each app topic tailored to a clinician audience and have additional drop-down menus on the app, such as *suggested homework* that the clinician can assign.

**Figure 2:**
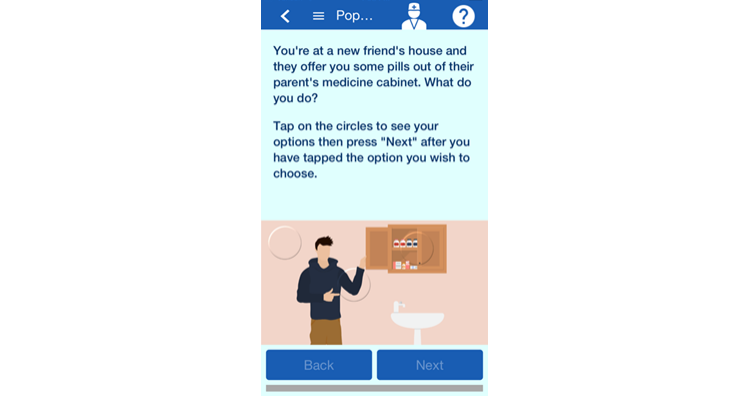
Trauma-Informed Prevention for Substance Use and Risky Sexual Behavior Make a Play substance use prevention activity screen example.

**Figure 3:**
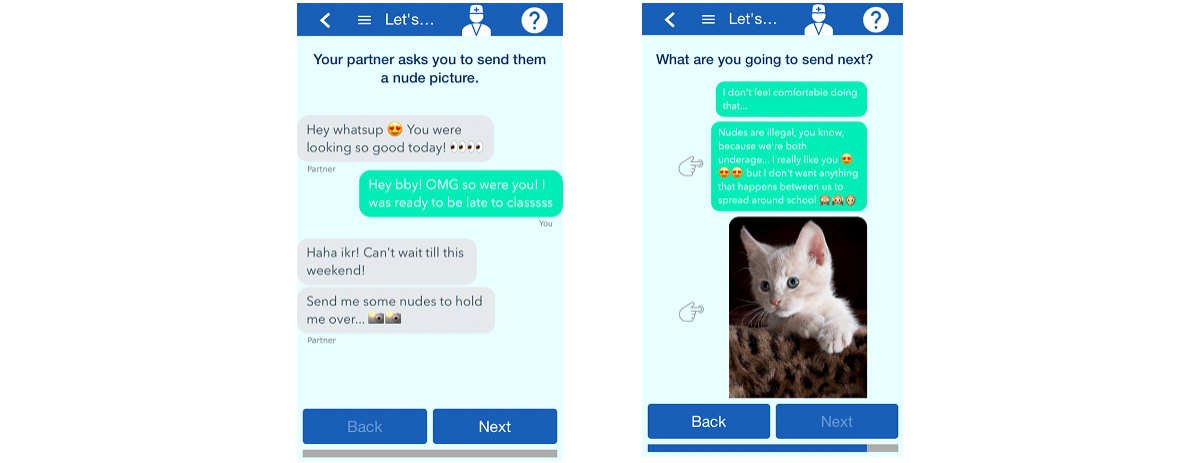
Trauma-Informed Prevention for Substance Use and Risky Sexual Behavior Make a Play risky web behavior prevention activity screen example.

In particular, the app was designed to map onto the psychoeducation, parenting, and enhancing safety treatment components of TF-CBT. Each topic of the app is introduced by a short video that explains the aims of the section as well as the directions needed to successfully complete the section. After the completion of each component activity (briefly described in this section), users are presented with feedback relevant to their performance on the current activity as well as resources (links to websites) to help further their education on the topic. Specifically, the *What Do You Know?* component is formatted as a multiple-choice trivia game that serves as a psychoeducational tool to educate trauma-exposed teenagers and their caregivers on the effects of drugs and alcohol. The *Myth Busters* component educates users on the common facts and myths about HIV and other STIs. This section was designed as a drag-and-drop game and includes a video that demonstrates how to perform an at-home HIV test kit. The inclusion of the video aimed to reduce negative connotations that may currently exist related to getting tested for HIV (eg, scary, involving needles, and time-consuming). The *Hot or Not* component was designed as a psychoeducational tool to aid adolescents in recognizing unhealthy behaviors within romantic relationships. This section comprises engaging videos, multiple-choice trivia, and feedback that is presented after response submission. A main goal was to highlight warning signs that may precede more obvious unhealthy behavior as trauma-exposed teenagers may be more likely to stay in unhealthy romantic relationships. *Make a Play* helps guide adolescents through tough yet common situations that teenagers may experience. This component consists of 5 activities that educate users on choices involving sending nude pictures, having unprotected sex, consuming alcohol, and being offered different types of drugs. These activities are presented as choose-your-own-adventure screenplays that allow adolescents to make different choices for each situation that is presented and view the results for their different decisions. Figure 2 illustrates an adolescent presented with the choice to accept or decline the offer of marijuana by a peer. The *Family* topic of the app presents the empirically supported *Family Check-Up* [69] in an engaging way, educating caregivers on the different components involved in positive parenting (eg, communication, encouragement, and supervision). This section consists of videos, text information, and quizzes. *Cyber Life* was created to aid in the education regarding safe web behaviors and choices. This component presents users with possible situations that they may encounter on the web and the choices they can make in a multiple-choice format. Each choice is followed by feedback on its positive aspects and the risks involved. The *Vision 25* topic is to be used as a guide in goal setting. This section helps users think about their current goals, what choices they can make to help them successfully accomplish their goals, and what choices they can make that may result in their goals becoming harder to reach. This section presents users with a road map (Figure 3) where they can choose different ages at which to set goals, providing guidance for both short- and long-term goals.

The TIPS platform was developed using AppBuilder, a content management system developed by our digital health team under our institution’s Clinical and Translational Science Award. AppBuilder includes a wide array of design templates and features to ensure that native iOS and Android apps can be created by researchers, program staff, and other team members who have no formal coding experience. Investigators and innovators use it to wireframe, pilot test, change and add content, and launch and evaluate mHealth apps without the extensive involvement of a technical development team. Mobile app developers often become involved in AppBuilder-based initiatives only on a strategic, limited basis, consistent with our goal of significantly shortening the timeline and costs associated with building mHealth apps.

### Usability Testing Overview

Usability testing is incorporated into technology development to improve user experience by measuring whether the user can successfully and effectively use the tool. Usability testing incorporating both qualitative and quantitative methods can also help identify barriers to task completion and examine areas that take the user off topic, create confusion, or decrease satisfaction [70]. The purpose of usability testing in this study was to obtain objective metrics and refine the final TIPS product before formal efficacy testing.

### Ethical Considerations

All procedures were approved by the Medical University of South Carolina Institutional Review Board (Pro00041527). Participants (ie, clinicians, caregivers, and teenagers) were given an information sheet that detailed the purpose of the research; procedures; risks, discomforts and benefits; costs and compensation; alternatives; and confidentiality. Participants provided verbal consent in lieu of written consent. Participant data were deidentified to preserve confidentiality. In addition, participants were compensated for their participation with US $30 in the form of a gift card, cash, or money order.

### Participants

Participants included mental health clinicians (11/32, 34%; 26/32, 82% female), trauma-exposed adolescents (11/32, 34%; 26/32, 82% female), and caregivers of adolescents (10/32, 31%; 25/32, 80% female). More specifically, with regard to inclusion criteria, clinicians were master’s or doctoral-level mental health providers in the area local to the study who were fully trained and experienced in TF-CBT and carried active child trauma caseloads. Adolescents were aged between 13 and 18 years, had experienced at least one traumatic event, were in the process of completing or had recently completed TF-CBT, and assented to participate (with caregiver consent). To be included, caregivers needed to have served for at least the previous 2 months in the role of primary caregiver of a teenager in treatment for PTSD who was in the process of completing or had recently completed TF-CBT. Among the clinicians, 72% (8/11) were aged between 25 and 34 years, whereas 27% (3/11) were aged between 35 and 44 years. The adolescents ranged in age from 13 to 18 (mean 15.25, SD 1.90) years, with most (9/11, 82%) falling between the ages of 13 and 17 years. Finally, caregivers ranged in age from 34 to 53 (mean 44.2, SD 6.65) years.

### Procedures

Participants (clinicians, adolescents, and caregivers) were recruited from community-based mental health centers that specialized in the treatment of traumatic stress and served multiple counties (including urban and rural areas) in a large city in the Southeast United States. There were 2 primary methods through which participants were recruited: (1) flyers were posted in and around the clinics with contact information for the study project coordinator and (2) potential participants were informed by clinic staff that they may qualify for a study and asked whether they would be interested in learning more. When potential participants responded positively, the study coordinator contacted them and provided a full description of the study, screened them to ensure that the inclusion criteria were met (see the previous section), and consented them to participate in a qualitative interview regarding the TIPS app and completion of the Website Analysis and Measurement Inventory (WAMMI; see the following section).

### Measures

The semistructured qualitative interview consisted of a study team member providing the participant with an iPad loaded with the TIPS app and walking the participant through each section. Following each section of the app, the interviewer asked several open-ended questions (eg, “First, tell me, what are you thinking when you look at this page?” “What do you like about this activity?” “What don’t you like about this activity?” and “How can we make this more interesting to teens?”). Follow-up probes were used to clarify the information provided whenever necessary.

The WAMMI [71] is a standardized 20-item assessment measure that captures users’ personal opinions on a given website’s ease of use. The items were slightly revised to refer to the application rather than the website. In the measure, users are asked to rate various aspects of their experience with the app (eg, content, navigation, and design) on a 5-point Likert scale from *strongly agree* to *strongly disagree*. Items are then scored to produce 5 subscales measuring the app’s *attractiveness* (level of visual interest of the app in terms of both function and information provision), *control* (app navigation ease), *learning* (users’ ability to easily understand the content of the app and learn what they expect to learn), *helpfulness* (usefulness and expected content and structure of the app), and *efficiency* (users’ ability to quickly find and do what interests them on the app). The WAMMI was developed using latent variable analysis, has high reliability, and reports standardized scores (eg, 50=average; 100=perfect) for the 5 aforementioned themes based on a reference database [72].

### Data Analysis Plan

#### Quantitative Approach

All descriptive analyses were performed using Stata (version 17; StataCorp LLC) [73]. The data were screened for outliers and impossible values. Group differences in the WAMMI items were compared using 2-tailed paired *t* tests.

#### Qualitative Approach

Data analysis consisted of a qualitative content analysis [74] informed by grounded theory [75], which is used to explore participants’ unique perspectives via the identification of themes and patterns that naturally emerge from the data and the systematic classification of these themes. Specifically, a 3-step inductive approach was used, which involves collecting and analyzing data without preconceived categories or theories. To analyze using this approach, each participant’s interview responses (ie, raw data) were carefully examined to develop a comprehensive codebook to capture all possible themes emerging from the data. The codebook was then used by 2 independent coders to code and analyze each participant’s responses to the interview questions [74,76]. Coders were able to apply more than one code to the participant responses if applicable. The interrater reliability for the double-coded interview responses was 86% and ranged from 82% to 93%. Interrater discrepancies were discussed and resolved by the 2 independent coders. Finally, themes were refined, merged, or divided into subthemes via collaborative discussions in multiple in-person meetings until a comprehensive codebook was developed. The NVivo software (version 11.1; Lumivero) was used for data management and analysis. The interviews were approximately 45 minutes in length and were audio recorded and transcribed.

## Results

### Technology and App Use Descriptive Information

Clinicians answered some descriptive questions about comfort using smartphones and apps and the benefits and drawbacks of using websites or apps in treatment. All clinicians (10/10, 100%) reported (1) being comfortable using smartphones and tablets and (2) that the primary benefit of using websites and mHealth app tools is that they engage teenagers in treatment and that they are readily accessible. Other benefits reported by clinicians included that apps are free and that teenagers feel comfortable using them. Drawbacks reported by clinicians included that it is difficult to get teenagers to stop using apps and that not all homes have Wi-Fi access.

Adolescents and caregivers were asked about their use of tablets and cell phones. Most adolescents (8/11, 73%) and caregivers (8/10, 80%) reported personally owning a tablet, and all adolescents (11/11, 100%) and caregivers (10/10, 100%) reported owning a cell phone. The primary uses of tablets by adolescents included accessing social media, playing games, watching television shows or movies, SMS text messaging, and completing schoolwork. Adolescents reported that they primarily used their cell phones for talking to friends, SMS text messaging, accessing social media, playing games, and listening to music. Caregivers reported that they primarily used their tablets for playing games, paying bills, surfing the internet, watching television shows and movies, school and work, accessing social media, and checking email. Caregivers primarily used their cell phones for talking to people, SMS text messaging, accessing social media, and surfing the internet.

### Qualitative Results

#### Overview

Through the individual interviews, valuable information about the usability and perceived effectiveness of TIPS was obtained from clinicians, adolescents, and caregivers. Four overarching themes, each with its own subthemes, emerged from the participants’ answers to the interview questions: (1) strength of app content, (2) suggestions about app content, (3) esthetics and usability, and (4) benefits to the patient and session implementation. Table 1 shows the app themes that emerged. Each is described in greater detail in the following sections, with representative quotes provided throughout for illustrative purposes.

**Table 1:** Trauma-Informed Prevention for Substance Use and Risky Sexual Behavior (TIPS) app themes and percentages yielded from the qualitative interviews^a^.

Theme		Responses, n (%)
**Strengths of app content**		64 (32.9)
	Comments regarding appropriateness of content for adolescents	27 (13.9)
	Content of modules	10 (5.2)
	Pictures or videos	1 (0.5)
	Content is entertaining or would keep adolescents’ attention	1 (0.5)
**Changes in or suggestions for app content**		90 (46.3)
	Make content more engaging	17 (8.8)
	Revise the video or picture content	9 (4.6)
	Suggestions for content topics to add	20 (10.3)
	Revise the language to make it more relatable to teenagers	22 (11.3)
	Allow content to be more individualized	15 (7.7)
**Esthetics and usability**		30 (15.5)
	Strengths regarding esthetics of the app	8 (4.1)
	Suggestions for esthetics of the app	17 (8.8)
	Dislikes regarding esthetics of the app	5 (2.6)
**Benefits to the patient and session implementation**		6 (3.1)
	Increases comfort of the adolescent in the session	5 (2.6)
	Would be more likely to use the TIPS app	2 (1)

^a^Themes that emerged from the responses of all users during the qualitative interviews (32 participants and 194 responses).

#### Theme 1: Strengths in the App Content

A total of 62% (20/32) of the participants discussed the strengths of the content of the TIPS app. Most strengths mentioned within this theme included comments about the appropriateness of the content for adolescents (21/32, 66%), the content of the modules (17/32, 53%), pictures or videos (13/32, 41%), or that the content was entertaining and would keep an adolescent’s attention (11/32, 34%). Other comments centered on language content, diversity of content, positive framing of information, or that the content differed by activity. Clinicians, adolescents, and caregivers all agreed that the content was very relevant to adolescents and used examples and language that adolescents could relate to. All 3 groups also discussed that the content was comprehensive and addressed issues often faced by adolescents. For example, clinicians commented the following:

I really like the feedback sessions when you give the examples. They’re very detailed, but not overly detailed.

I think the language is really accessible to most kids.

Examples of strengths stated by adolescents include the following:

The videos are funny. I definitely like those.

I actually think that the text itself, that was pretty cool how y’all made it like the iPhone with all the emojis and stuff. And it is kind of relatable to people my age, at least, text. And the responses to it were...like sending the cat picture, that’s definitely something people would do if they don’t feel comfortable and they’re trying to make it funny.

Strengths mentioned by caregivers included the following:

I think the Let’s talk about Sext [video] needs to come as soon as you put the phone in their hand. Plain and simple, right off the rail. It’s not like there’s going to be a certain age, it’s like having the talk about the birds and the bees.

Man, I liked the points they’re making. You know, I think they’re absolutely making the right points with, you know, being present, you know, when your kids are around. Knowing your kids’ friends, knowing where they are, surprise phone calls. I like all that stuff.

#### Theme 2: Changes in or Suggestions for App Content

A total of 59% (19/32) of the participants discussed changes or suggestions regarding the TIPS app content. Most comments within this theme included making content more engaging (22/32, 69%), revising the video or picture content (22/32, 69%), suggestions for content topics to add (17/32, 53%), revising the language to make it more relatable to teenagers (15/32, 47%), and allowing the content to be more individualized (8/32, 25%). Other comments included language suggestions, adding more outside resources, increasing cultural sensitivity, clarifying content, improving instructions, and removing potentially triggering content. Although the specific suggestions differed among the clinicians, adolescents, and caregivers, the feedback overlapped in that adding more interaction and individualizing some of the content would help improve the app. Some examples of comments made by clinicians include the following:

I’ve seen a lot of kids get through school that can’t read, so try to look at all factors. Maybe some more visual aids along with the words.

Some statements made by adolescents included the following:

Add a game or something. I don’t know what type of game, but a game that you can play for the answer.

Maybe even include something about rape in a relationship, because a lot of people that I’ve talked to, they don’t think that you can be raped in a relationship.

Comments made by caregivers included the following:

You know, make it a little bit more interesting. If it could be made more interactive.

A lot of kids don’t have parents. They’re already living with a family member, or in foster care. Another line to put in there somewhere is, talk to someone you trust...doesn’t have to be a parent.

#### Theme 3: Esthetics and Usability

A total of 25% (8/32) of the participants discussed the esthetics and usability of the TIPS app. The most common comments within this theme involved strengths regarding the esthetics of the app (15/32, 47%), suggestions for the esthetics of the app (13/32, 41%), and dislikes regarding the esthetics of the app (9/32, 28%). Participants also made a few comments about usability, including suggestions and strengths regarding the functionality of the app. All 3 of the groups (clinicians, adolescents, and caregivers) made suggestions about the esthetics, which mostly comprised suggestions to change the font, color, or pictures within the app. Of all the groups, adolescents were the most positive about the esthetics and usability of the app. Some specific comments made by clinicians included the following:

Maybe use highlight and change the color or something to make it clear. I think that the progress bar at the bottom is so simple. It stays out of the way, so you’re not sacrificing real estate.

[I like] the progress bar on the bottom. It’s a nice touch because then I don’t have to keep wondering how many more questions. I can see that I’m almost done, so that helps me not get frustrated, especially for a teen.

Some examples of statements made by adolescents include the following:

The activities are very easy to operate.

I think it’s a good font size, I wear glasses, and I didn’t have to squinch my eyes to see. I also like how the page is light blue, and it’s dark blue for the words to stick out. So you’ll be able to look at it. That’s one thing I like about it.

Specific comments made by caregivers included the following:

I like the subtitles on the bottom, because I feel like you sometimes get lost in the words, so I like that they’re there.

I like the bright colors. The bright colors catch my eye.

#### Theme 4: Benefits to the Patient and Session Implementation

A total of 19% (6/32) of the participants discussed how the TIPS app benefits the patient or made comments about session implementation. Most comments within this theme included that the TIPS app increases the comfort of the adolescent in the session (10/32, 31%) or that clinicians would be more likely to use the TIPS app with particular patients (10/32, 31%), such as female individuals or sexually active teenagers. Other comments in this theme included that the app makes the session more interactive, increases engagement for teenagers, is easy to incorporate into the session, and allows the clinician to modify or select content based on patient needs. Some examples of comments made by clinicians include the following:

This is good for me, too, the way I’m able to give feedback to the patient, or at least talk about it, cuz once it’s there, okay, I get it, and I can explain it to them, so I really like it.

Comments made by adolescents included the following:

The app makes it easier because it’s just awkward to talk about these things.

Some statements from caregivers included the following:

I think for this exercise as far as getting them to wrap their brain around where they really have established rapport with you, it’s probably a nice way of doing that where they don’t really have to look you in the eye and tell you about what they want.

### Quantitative Results

The results of the WAMMI across the 3 groups are shown in Table 2. Responses across the groups indicated that all participant groups viewed the app favorably. In general, clinicians tended to report the most critical responses to the app. Overall, adolescents found the app (relatively) easier to use and understand compared with clinicians and caregivers. Clinicians had a greater propensity to report the app having annoying features compared with caregivers and adolescents. All clinicians (11/11, 100%) and caregivers (10/10, 100%) and 64% (7/11) of adolescents selected *somewhat agree* or *strongly agree* for the following statement: “This app has much that is of interest to me.” All adolescents (11/11, 100%) somewhat agreed or strongly agreed with the following statements: “I can quickly find what I want on this app,” “Using this app for the first time is easy,” and “Everything on this app is easy to understand.”

**Table 2:** Website Analysis and Measurement Inventory (WAMMI) responses regarding the Trauma-Informed Prevention for Substance Use and Risky Sexual Behavior app by group^a^.

Statement	Clinicians (n=11)		Caregivers (n=10)		Adolescents (n=11)	
	Mean (SD)	Median (range)	Mean (SD)	Median (range)	Mean (SD)	Median (range)
This app has much that is of interest to me.	1.73 (1.19)	1 (1-5)	1.20 (0.42)^b^	1 (1-2)	2.36 (0.81)^b^	2 (1-4)
It is difficult to navigate this app.	2.91 (1.04)^c^	2 (2-4)	3.90 (1.79)	5 (1-5)	4.64 (0.92)^c^	5 (2-5)
I can quickly find what I want on this app.	2.36 (1.03)^d^	2 (1-4)	1.40 (0.70)^d^	1 (1-3)	1.45 (0.52)	1 (1-2)
This app seems logical to me.	1.55 (1.21)	1 (1-5)	1.50 (1.27)	1 (1-5)	1.36 (0.67)	1 (1-3)
This app needs more introductory explanations.	3.45 (0.93)	4 (2-4)	3.80 (1.40)	4 (1-5)	3.82 (1.54)	4 (1-5)
This app is very attractive.	2.55 (1.37)	2 (1-5)	1.90 (1.29)	1 (1-4)	2.36 (1.03)	2 (1-4)
I feel in control when I’m using this app.	2.09 (1.04)	2 (1-4)	1.50 (0.97)	1 (1-4)	1.73 (0.79)	2 (1-3)
This app is too slow.	4.0 (1.0)	4 (2-5)	3.90 (1.60)	5 (1-5)	4.18 (0.98)	4 (2-5)
This app helps me find what I am looking for.	2.09 (0.94)	2 (1-4)	1.70 (1.06)	1 (1-4)	1.73 (0.65)	2 (1-3)
Learning to find my way around this app is a problem.	3.45 (1.04)^b^	4 (2-5)	4.30 (1.34)	5 (1-5)	4.73 (0.47)^c^	5 (4-5)
I don’t like using this app.	4.09 (1.58)	5 (1-5)	4.80 (0.42)	5 (4-5)	4.09 (1.04)	4 (2-5)
I feel efficient when I’m using this app.	2.27 (1.19)	2 (1-4)	1.60 (0.97)	1 (1-4)	2.09 (1.14)	2 (1-5)
It is difficult to tell if this app has what I want.	3.18 (1.47)	3 (1-5)	3.90 (1.66)	5 (1-5)	3.72 (1.19)	4 (2-5)
Using this app for the first time is easy.	2.64 (1.21)^c,d^	2 (1-4)	1.50 (1.27)^d^	1 (1-5)	1.45 (0.52)^c^	1 (1-2)
This app has some annoying features.	2.73 (1.49)^c,d^	2 (1-5)	4.40 (1.07)^d^	5 (2-5)	4.18 (1.17)^c^	5 (2-5)
Remembering where I am on this app is difficult.	3.18 (1.08)^c^	3 (2-5)	3.70 (1.70)^b,c^	4.5 (1-5)	4.82 (0.40)^b,c^	5 (4-5)
Using this app is a waste of time.	4.45 (0.93)	5 (2-5)	4.90 (0.32)	5 (4-5)	4.64 (0.92)	5 (2-5)
I get what I expect when I click on things on this app.	2.00 (1.10)	2 (1-4)	1.80 (1.48)	1 (1-5)	1.55 (0.52)	2 (1-2)
Everything on this app is easy to understand.	2.45 (1.13)^c,d^	2 (1-4)	1.30 (0.48)^d^	1 (1-2)	1.36 (0.50)^c^	1 (1-2)

^a^WAMMI responses among all users interviewed, including clinicians, adolescents, and caregivers*.* Each item was scored on a Likert scale (1=*strongly agree*, 2=*somewhat agree*, 3=*neutral*, 4=*somewhat disagree*, and 5=*strongly disagree*). Lower numbers indicate greater agreement with the statement.

^b^Significant differences between the caregiver and adolescent groups (*P*<.05).

^c^Significant differences between the clinician and adolescent groups (*P*<.05).

^d^Significant differences between the clinician and caregiver groups (*P*<.05).

## Discussion

### Principal Findings

As of October 2021, a national state of emergency in child mental health has been jointly declared by the American Academy of Pediatrics, American Academy of Child and Adolescent Psychiatry, and Children’s Hospital Association because of the rising rates of behavioral health problems coupled with a limited and overburdened mental health workforce [52]. To address this public mental health crisis, it is critical to enlist a broad and creative range of approaches, including those that leverage mHealth tools, to implement empirically supported content and interventions that target the significant drivers of this state of emergency. Also critical to this crisis is the need for more robust prevention of substance use problems (including opioid overdose and opioid use disorders) and HIV and other STI acquisition (particularly among young people at the highest risk for new HIV acquisition) [77,78], which is signaled by every public health indicator. The goal of this paper was to describe the development and usability testing of an mHealth app tool that collectively targets the aforementioned problems (ie, high prevalence of trauma-related mental health problems, substance use disorder, and opioid overdose and new HIV acquisition risk, as well as limited resources and opportunities dedicated to implementing substance use prevention and training clinicians in doing so).

The primary overall finding from the usability testing of the app leveraging a mixed methods approach was that the TIPS app was perceived by all 3 user types to be a highly usable mHealth tool to be implemented during the course of TF-CBT. Qualitative data collected from the usability testing of the app yielded positive feedback from clinicians, adolescents, and caregivers, and the quantitative data (ie, responses to the WAMMI) concurred with the qualitative findings (eg, 27/32, 84% of all users agreed or strongly agreed that “The app has much of interest to me”). However, more generally, the results of this study provide valuable insights into the use of technology and apps in adolescent mental health treatment. Clinician responses illustrated that they generally felt comfortable using smartphones and tablets and believed that these mHealth tools yielded benefits of engagement and accessibility for their adolescent clients. Moreover, the fact that all clinicians reported comfort with using these devices suggests a high level of digital readiness among this workforce in need of resources and support, which is a shared sentiment among other clinicians [79]. Despite this positive feedback, clinicians also identified potential drawbacks related to apps and mHealth tools, including the challenge of teenagers potentially overusing or being distracted by apps and issues related to limited wireless internet access in some homes. Indeed, equitable access to technology is essential when developing, evaluating, and implementing mHealth tools as augmentations to mental health interventions.

Specific to the interview responses, 4 overarching themes related to the TIPS app’s usefulness and effectiveness emerged. The first theme, *strengths of the app content*, highlights the positive aspects of the app, such as content tailored well to an adolescent audience, modules that are viewed as engaging, and relatable language. These strengths are crucial for maintaining adolescent clients’ attention and fostering meaningful interactions during trauma treatment, particularly when talking about what may be perceived as sensitive topics (eg, sexual decision-making). The second theme, *changes in or suggestions for app content*, points to the need for interactivity, revisions in video and picture content, and the inclusion of important topics such as consent and rape in relationships. Echoing findings of previous studies, these suggestions underscore the importance of user input and continuous improvement and adaptation of app content to meet evolving needs [80]. This also highlights the importance of using tools that allow for efficient and inexpensive minor edits (eg, to language and images) to mHealth apps when possible, as was done using the AppBuilder platform for the TIPS app. The third theme, *esthetics and usability*, emphasizes the significance of the app’s design and functionality, with users offering both praise and suggestions for improvement. Beyond further underscoring the importance of having the capacity to revise content within an app as part of the iterative app development process, these praise interview responses also help inform implementation strategies, indicating what end users like most and find most engaging about the app. For example, regarding TIPS, the *Make a Play* activity emerged as a favorite (eg, the opioid pill activity and *Let’s talk about Sext*), and it may be an activity to highlight when first explaining the app to a clinician or adolescent client (eg, engaging). Finally, the fourth theme, *benefits to the patient and session implementation*, highlights the app’s potential to enhance adolescent and caregiver comfort during sessions and improve engagement, particularly for sensitive topics. It also suggests that clinicians are more likely to use the app with specific patient demographics, such as sexually active teenagers.

Regarding one of the clinicians’ comments that it can be difficult to get teenagers to stop using apps, it is important to note that a detailed implementation manual for the TIPS app is provided to clinicians when they are trained in how to use the app. Specifically, they are guided on how to structure the time spent on activities on the app, which occurs in the context of a TF-CBT treatment session. Thus, TF-CBT clinicians are able to contain adolescents’ use of the TIPS app in sessions.

The quantitative results, presented in Table 1, reinforce the positive reception of the TIPS app across all participant groups. Adolescents in particular found the app less difficult to use and more understandable compared with clinicians and caregivers, but clinicians expressed some critical feedback, including minor annoyance with certain features. Notably, all clinicians and caregivers, along with most adolescents, expressed a strong interest in the app’s content. In addition, adolescents found the app easy to navigate and understand, suggesting that user-friendliness is a key factor in their engagement with digital mHealth tools [81]. Overall, these quantitative findings align with the qualitative feedback, highlighting the promising utility of the TIPS app for clinician implementation among adolescents and caregivers receiving TF-CBT while also emphasizing areas for improvement.

A strength of the TIPS tool is that it offers a platform that is telehealth compatible. The past several years have underscored the value of ensuring access to mental health treatment and risk behavior prevention through telehealth strategies. In addition to the benefits of breaking down geographical barriers, the integration of mHealth tools such as TIPS with telehealth helps promote confidential and convenient interactions for patients with mental health clinicians, fostering a sense of privacy and comfort that is crucial for therapy [82,83]. This may be particularly helpful when addressing sensitive topics such as trauma, substance use, and HIV. Ultimately, the synergy between mHealth tools and telehealth in behavioral care represents a transformative shift toward prevention-focused holistic and inclusive mental well-being support.

### Limitations

The primary limitation of this study is that it is limited in scope to usability testing with a small sample of clinicians, adolescents, and caregivers. To establish the feasibility and efficacy of the app in reducing the risk of substance use and risky sexual behavior, a fully powered RCT is necessary to compare TF-CBT implementation with and without the inclusion of the app, including assessment time points that follow youth and their caregivers over time. Although this study informs possible revisions to the app and suggests that clinicians, adolescents, and caregivers will respond positively to its inclusion in trauma-focused treatment, the efficacy trial will ultimately reveal whether indeed the app is able to make a dent in the youth mental health state of emergency and help eliminate some of the burden on clinicians in implementing a prevention curriculum.

### Conclusions

In conclusion, this study demonstrates the positive reception of technology—and the TIPS app in particular—in adolescent trauma-focused treatment, with clinicians, adolescents, and caregivers recognizing the benefits of engagement, accessibility, and user-friendliness of this novel mHealth tool. The qualitative themes shed light on the strengths of the app’s content, areas for improvement, esthetics, and usability as well as its potential to enhance adolescents’, caregivers’, and clinicians’ experiences during TF-CBT sessions. These findings underscore the importance of the ongoing development and refinement of digital tools in mental health care—including those that can be integrated into telehealth mental health care delivery—to better meet the evolving needs of trauma-affected adolescents and their caregivers.

## Abbreviations

mHealth: mobile health
PTSD: posttraumatic stress disorder
RCT: randomized controlled trial
STI: sexually transmitted infection
TIPS: Trauma-Informed Prevention for Substance Use and Risky Sexual Behavior
TF-CBT: trauma-focused cognitive behavioral therapy
WAMMI: Website Analysis and Measurement Inventory

## Data Availability

The data are available from the study principal investigator (CKD) upon request.
